# *Babesia* spp. and *Anaplasma phagocytophilum* in questing ticks, ticks parasitizing rodents and the parasitized rodents – Analyzing the host-pathogen-vector interface in a metropolitan area

**DOI:** 10.1186/1756-3305-5-191

**Published:** 2012-09-05

**Authors:** Cornelia Silaghi, Dietlinde Woll, Dietmar Hamel, Kurt Pfister, Monia Mahling, Martin Pfeffer

**Affiliations:** 1 Comparative Tropical Medicine and Parasitology, Ludwig-Maximilians-Universität München, Munich, Germany; 2 Institute of Animal Hygiene and Veterinary Public Health, University Leipzig, Leipzig, Germany; 3 Statistical Consulting Unit, Department of Statistics, Ludwig-Maximilians-Universität München, Munich, Germany

**Keywords:** *Babesia* spp, *Anaplasma phagocytophilum*, *Ixodes ricinus*, *Dermacentor reticulatus*, Bank vole, Yellow-necked mouse, Recreational area, Host survey, Vector-host relation

## Abstract

**Background:**

The aims of this study were to evaluate the host-tick-pathogen interface of *Babesia* spp. and *Anaplasma phagocytophilum* in restored areas in both questing and host-attached *Ixodes ricinus* and *Dermacentor reticulatus* and their small mammalian hosts.

**Methods:**

Questing ticks were collected from 5 sites within the city of Leipzig, Germany, in 2009. Small mammals were trapped at 3 of the 5 sites during 2010 and 2011. DNA extracts of questing and host-attached *I. ricinus* and *D. reticulatus* and of several tissue types of small mammals (the majority bank voles and yellow-necked mice), were investigated by PCR followed by sequencing for the occurrence of DNA of *Babesia* spp. and by real-time PCR for *A. phagocytophilum*. A selected number of samples positive for *A. phagocytophilum* were further investigated for variants of the partial *16S rRNA* gene. Co-infection with *Rickettsia* spp. in the questing ticks was additionally investigated.

**Results:**

4.1% of questing *I. ricinus* ticks, but no *D. reticulatus*, were positive for *Babesia* sp. and 8.7% of *I. ricinus* for *A. phagocytophilum*. Sequencing revealed *B. microti*, *B. capreoli* and *Babesia* spp. EU1 in Leipzig and sequence analysis of the partial *16S RNA* gene of *A. phagocytophilum* revealed variants either rarely reported in human cases or associated with cervid hosts. The statistical analysis revealed significantly less ticks infected with *A. phagocytophilum* in a city park in Leipzig as compared to the other sampling sites. *A. phagocytophilum*-DNA was detected in 2 bank voles, DNA of *B. microti* in 1 striped field-mouse and of *Babesia* sp. EU1 in the skin tissue of a mole. Co-infections were detected.

**Conclusion:**

Our results show the involvement of small mammals in the natural endemic cycles of tick-borne pathogens. A more thorough understanding of the interactions of ticks, pathogens and hosts is the essential basis for effective preventive control measures.

## Background

Tick-borne disease agents form complex life cycles involving an arthropod and vertebrate host. The complexity is enhanced by the diversity of hosts in different biotopes, which depends on factors like type of vegetation, climate and/or human influence, such as restoration of former industrial sites which leads to the development of new biotopes. Thus, on the one hand, new habitats for plants and animals including ticks, and on the other hand, new interfaces for humans and nature are created.

In contrast to *Ixodes ricinus*, the most common tick in Europe, *Dermacentor reticulatus* has a focal distribution. *I. ricinus* is an important vector for pathogens such as *Borrelia burgdorferi* sensu lato and the tick-borne encephalitis virus, but also for so-called emerging pathogens like *Anaplasma phagocytophilum* and *Babesia* spp. [1]. *D. reticulatus* as well serves as a vector for pathogens of human and veterinary concern, e. g. for *Rickettsia raoultii* (causing Tick-Borne Lymphadenopathy - TIBOLA) and *Babesia canis canis* (one of the causative agents of canine babesiosis) [2,3]. Canine babesiosis can be a mild to serious disease in dogs, leading even to organ failure and death [2]. In the USA, human babesiosis is primarily caused by *B. microti*, in Europe by *B. divergens* and few cases caused by *B. microti* and *Babesia* sp. EU1 have been described [4-7]. Whereas ticks as well as cattle and roe deer can serve as a reservoir for *B. divergens* and *Babesia* sp. EU1 due to transovarial transmission, *B. microti* is not transmitted transovarially and small mammals are the reservoir [8-11]. *A. phagocytophilum* causes granulocytic anaplasmosis in dogs, horses and humans [12]. Transovarial transmission of *A. phagocytophilum* has not been proven in *I. ricinus,* and several mammalian species are under discussion as reservoir hosts [12]. Previous infection rates of *Babesia* spp. and *A. phagocytophilum* detected in questing *I. ricinus* in Germany are listed in Tables 1 and 2. Comparison of *Babesia* prevalences from previous studies shows that infection rates are generally lower, by a factor of 5 to 10, in the South of Germany (Table 1). Both babesiosis and granulocytic anaplasmosis are zoonotic diseases with a natural enzootic cycle involving the agent itself, the tick and the host. For both tick species and disease agents, humans can be considered accidental hosts acquiring the respective infection during interaction with the natural enzootic cycle. In this context, recreationally used urban and suburban green areas are intriguing habitats due to the strong influence that humans exert on them and thus, these areas should be given special consideration with regard to host-pathogen-vector interactions. We have previously shown high prevalences of spotted fever group rickettsiae in questing *I. ricinus* and *D. reticulatus* in recreationally used renatured areas [13]. The aims of the present study were the evaluation of the tick-host-pathogen interface in recreational areas in Leipzig, Germany. Therefore we attempted (i) to identify *Babesia* infections in questing and host-attached *I. ricinus* and *D. reticulatus* populations, (ii) to identify the infection rate with *A. phagocytophilum* in questing and host-attached *I. ricinus*, (iii) to identify possible small mammalian reservoir hosts in the investigated areas and (iv) to evaluate co-infections with *Rickettsia* spp. in the same questing ticks which had been investigated for *Rickettsia* spp. in a previous study [13]. 

**Table 1:** **Previously identified infection rates of *Babesia* spp. in questing *Ixodes ricinus * in Germany**

**Year of tick collection**	**Location in Germany**	**No. of ticks**	**Average prevalence in%**	**Detected species**	**Method**	**Target gene**	**Reference**
2003	North	127	0	n.a.	Reverse Line Blot	*18S rRNA*	[82]
2006-2007	Central	1,000	5.0	*B. microti* > *B. divergens*, *Babesia* spp.	Conventional PCR	*18S rRNA*	[83]
2007	Central	196	10.7	*B. divergens/B. microti*	Conventional PCR	*18S rRNA*	[79]
1999-2001	South	3,113	1.0	*B. divergens* > *B. microti*	Conventional PCR	*18S rRNA*	[78]
2003-2004	South	625	1.3	Piroplasmidae (not further specified)	Real-time PCR	*18S rRNA*	[84]
2009-2010	South	6,593	0.4	*Babesia sp. EU1* > *B. divergens*, *B*. *divergens/capreoli*, *B. gibsoni*-like	Conventional PCR	*18S rRNA*	[18]

n.a., not applicable.

**Table 2:** **Previously identified infection rates with *Anaplasma phagocytophilum * in questing *Ixodes ricinus* in Germany**

**Year of tick collection**	**Location in Germany**	**No. of ticks**	**Average prevalence in%**	**Method**	**Target gene**	**Reference**
2003	North	127^b^	3.9	Reverse Line Blot	*16S rRNA*	[82]
2005	North	1,646^c^	3.2	Real-time PCR	*Msp2*^*a*^	[85]
2003	Central	305^c^	2.3	Conventional PCR	*16S rRNA*	[86]
2006-2007	Central	1,000^c^	5.4	Conventional PCR	*16S rRNA*	[73]
2007	Central	196^c^	0	Conventional PCR	*16S rRNA*	[79]
1999	South	492^c^	1.6	Conventional PCR	*16S rRNA*	[87]
1999-2001	South	5,424	1.9	Nested PCR	*16S rRNA*	[78]
2002	South	1,963	2.8	Nested PCR	*16S rRNA*	[88]
2003-2004	South	625	4.5	Real-time PCR	*Msp2*^*a*^	[84]
2006	South	2,862^c^	2.9	Real-time PCR	*Msp2*^*a*^	[29]
2009-2010	South	5,569^c^	9.0	Real-time PCR	*Msp2*^*a*^	[53]
1998 -2010	Total	20,209	4.3			

^a^ multicopy target.

^b^ only nymphs.

^c^ study incl. nymphs.

## Methods

### Sampling sites and questing tick collection

**Figure 1:**
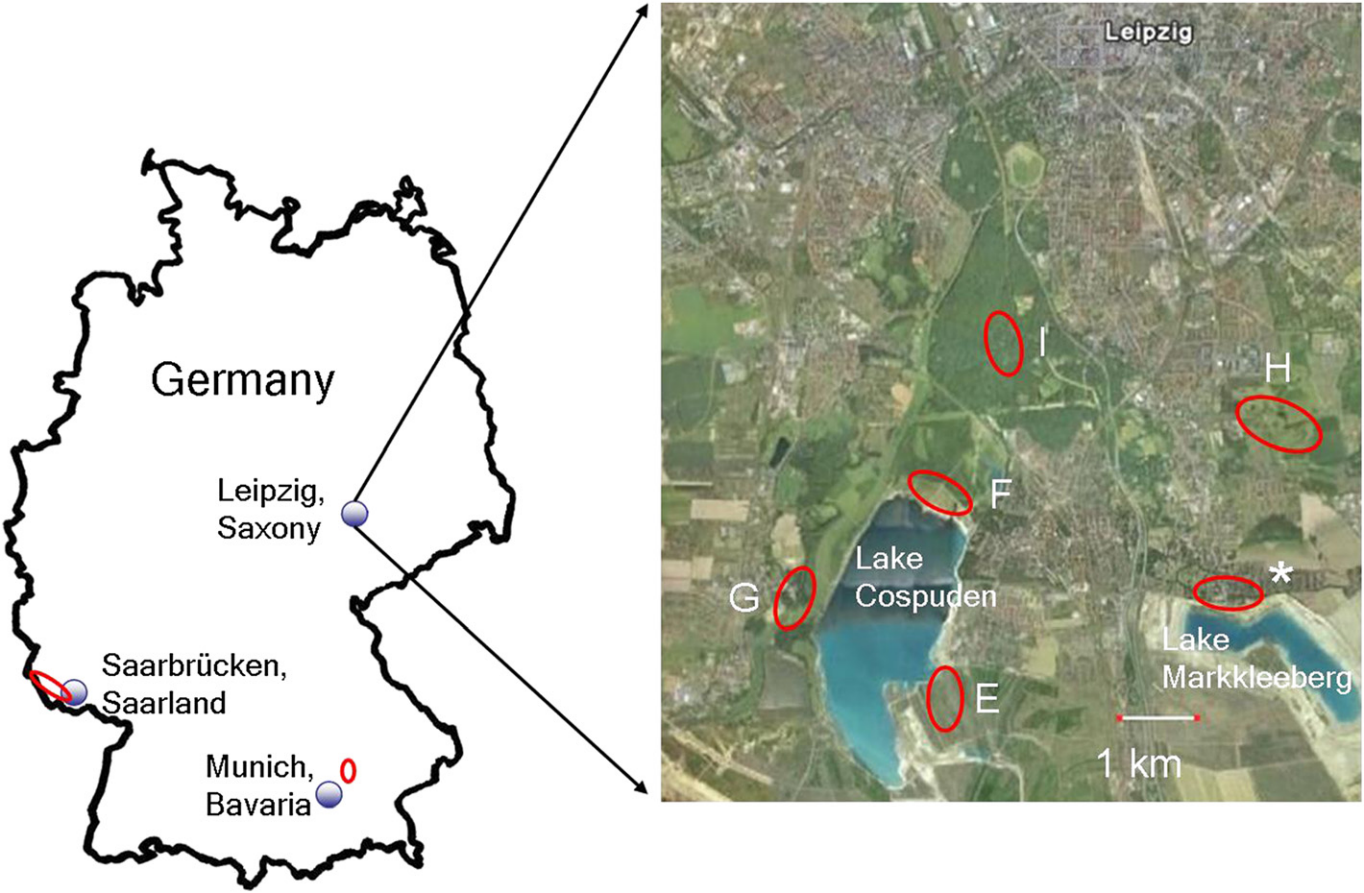
Sampling sites in the Saarland, Bavaria and sites E-I in the city area of Leipzig are indicated with red rings; the * marks the site where additional animals were caught by a cat.

Questing ticks were collected with the flagging method in 5 sites (E-I) in Leipzig (Saxony, Germany), all located within the city or close to suburban residential areas (Figure 1). Sites E-G are part of the “Leipziger Neuseenland” ( http://www.leipzigerneuseenland.de/), a restored area of former pit holes of a brown coal surface mining site. Brown coal was mined till 1990 and flooding of the area started in 1993. When fully flooded, the area will contain 18 lakes. Ticks were collected around Lake Cospuden (Sites E-G; fully flooded since 2000; 436 ha in size). Site E is located on the Eastern, Site F on the Northern and site G on the Western shore. The area is popular for hiking, jogging, cycling and dog walking.

Further ticks were collected in the recreational city park “Lößnig-Dölitz” (site H; ( http://www.leipzig.de/de/buerger/freizeit/leipzig/parks/park_loessnig/), containing several small ponds. This park is located in an area which was used for underground brown coal mining up to 1959 and was also created by renaturation in the 1980s . To the North it borders to the city of Leipzig and to the South, where a renatured waste disposal mountain is located, it merges directly into forest areas. Site I is located in one of the largest riparian forest areas in Germany, within the city of Leipzig ( http://www.leipziger-auwald.de). A 42 ha game park ( http://www.leipzig.de/de/buerger/freizeit/leipzig/parks/wildpark/wissen/) with about 250 animals (amongst them roe deer, red deer, fallow deer, wild boar, mustelids and several bird species) is situated within this forest.

For comparison, one site in Bavaria, located 20 km north of the city of Munich in a riparian forest on the Eastern banks of the river Isar was chosen. The area is popular for hiking, biking and dog walking.

Ticks were collected in April and September/October 2009 and were stored as described [13]. Further *I. ricinus* ticks were available from a previous study [14] from an area in the Saarland of altogether 150 km^2^ west of Saarbrücken (collection from March to September 2008). That previous study was concerned primarily with *D. reticulatus* and the *I. ricinus* were collected but kept aside.

### Trapping of small mammals

Small mammals were trapped at 3 out of 5 sites in Leipzig with Sherman live traps baited with apple pieces (August 2010 sites E and I; October 2010 / March 2011 site H; June 2011 sites E, H, I). Usually twenty traps per site were placed along natural structures or directly in front of holes in the ground. The number of traps per night never exceeded 75 and they all were checked twice daily. Captured animals were anesthetized with CO_2_ exposure and killed humanely according to the German Animal Protection Act after blood was drawn by heart puncture. After recording trapping site, species, gender, and health status, rodents were dissected under BSL-2 conditions in the laboratory and stored at −80°C. Ticks were collected from the small mammals and stored at −80°C separately for each individual animal until species identification.

### DNA extraction

Species identification of ticks was carried out with standard taxonomic keys [15-17] and DNA was extracted from the ticks as described [13] and from tissues and body liquids with the Qiagen DNA Mini Kit (Qiagen, Hilden, Germany) according to the manufacturer’s instruction for either animal tissue or blood. Tissue lysis was carried out over night at 56°C in a thermomixer (Eppendorf, Hamburg, Germany). Quality and quantity of extracted DNA were tested with a spectrophotometer (NanoDrop ND-1000, Erlangen, Germany).

### PCR methods

Detection of *Babesia* spp.-DNA in all tick and small mammal samples was carried out with a conventional PCR targeting the *18S rRNA* gene followed by gel electrophoresis as described [18,19]. Detection of *A. phagocytophilum* was carried out in *I. ricinus* ticks only and in the small mammals with a real-time PCR as described [20,21]. For a subsample of the *A. phagocytophilum* positive samples, a nested PCR targeting a 497 bp region of the *16S rRNA* gene was performed as previously described [22,23]. Genomic DNA from *A. phagocytophilum* cell culture and from *B. microti* from continuous culture in BALB/c mice (courtesy of Prof. Dr. E. Siński) were used as positive controls and molecular grade water as negative controls and were included in each PCR run.

### Sequence analysis

PCR products of the partial *Babesia* spp. *18S rRNA* gene and of the partial *16S rRNA* gene of *A. phagocytophilum* were purified with the QIAquick PCR Purification Kit (Qiagen, Hilden, Germany) and sequenced bidirectionally at Eurofins MWG Operon (Martinsried, Germany). The analysis of the obtained sequences was performed as described [24].

### Co-infections and statistical analysis of questing tick results

Exact confidence intervals of the prevalences (CI 95%) were computed with the Clopper and Pearson method. For pooled samples the confidence interval was calculated for the minimal infection, taking into account the nymphal pools and assuming that only one of the nymphs in one pool was infected.

Differences in the infection rates with *Anaplasma phagocytophilum*, *Babesia* spp. and *Rickettsia* spp. (data in the same questing ticks from a previous study [13]) between sex of ticks, collection sites and region were computed with logistic regression (generalized linear models with binominal distribution and logit link) using R 2.13.1 [25]. Separate models had to be computed to investigate the effect of collection sites and region. Moreover, separate models were used for the two species. However, a variable containing the information about the developmental stage and the sex of ticks was included as covariate in all models. P-values <0.05 were regarded as statistically significant. Co-infections of the questing ticks with *Rickettsia* spp. were investigated as data on this pathogen in the same ticks was available from a previous study [13]. As the trapping of small mammals took place at irregular intervals, no statistical analysis was attempted for the infestations of small mammals with ticks.

### GenBank accession numbers

Sequences from this study were deposited in GenBank [accession nos. *B. microti*: JQ886033- JQ886058, *Babesia* spp. EU1: JQ886024-JQ886032, *B. divergens*: JQ886059-JQ886060, *B. capreoli*: JQ886061, *Hepatozoon* spp.: JQ886023, *Sarcocystis* spp.: JQ886022, *A. phagocytophilum*: JQ886009 - JQ886021].

## Results

### Questing tick collection

A total of 1.256 *I. ricinus* (506 females, 519 males, 231 nymphs) and 814 *D. reticulatus* (503 females, 311 males) were collected in recreational areas in Leipzig (Sites E-I; Table 3). At the comparative site in Bavaria, 331 *I. ricinus* (109 females, 159 males, 63 nymphs) and 153 *D. reticulatus* (102 females, 51 males) were collected. From the Saarland, 115 *I. ricinus* (43 females, 36 males, 36 nymphs) were available [13,14]. DNA was successfully extracted from 804 *D. reticulatus* in Leipzig and 135 *D. reticulatus* in Bavaria. DNA from only a part of the *I. ricinus* was extracted (for detailed numbers, see Table 4). 

**Table 3:** Results of the questing tick collection at recreational areas of Leipzig in spring and autumn 2009

	**April**		**September/October**	
**Site**	***I. ricinus***	***D. reticulatus***	***I. ricinus***	***D. reticulatus***
E	447	293	3	207
F	n. c.	n. c.	20	51
G	n. c.	n. c.	21	191
H	515	19	29	53
I	221	0	n. c.	n. c.
Total	1183	312	73	502

n. c., no collection was carried out.

**Table 4:** **Infection rates with *Babesia* spp. and *Anaplasma phagocytophilum* in ticks from recreational areas within the city limits of Leipzig, Saxony and at comparative sites in Bavaria and the Saarland**

	***I. ricinus***												***D. reticulatus***					
					**No. positive (%)**											**No. positive (%)**		
	**Total no. investigated**				**Babesiaspp.**				**A. phagocytophilum**				**Total no. investigated**			**Babesiaspp.**		
Site	F	M	N	**Total**	F	M	N	**Total**	F	M	N	**Total**	F	M	**Total**	F	M	**Total**
E	45	56	6	**107**	0 (0)	4 (7.1)	1 (16.7)	**5 (4.7)**	7 (15.6)	5 (8.9)	0 (0)	**12 (11.2)**	309	190	**499**	0 (0)	0 (0)	**0 (0)**
F	6	4	10	**20**	0 (0)	0 (0)	0 (0)	**0 (0)**	0 (0)	0 (0)	0 (0)	**0 (0)**	29	21	**50**	0 (0)	0 (0)	**0 (0)**
G	6	2	13	**21**	0 (0)	0 (0)	0 (0)	**0 (0)**	0 (0)	0 (0)	0 (0)	**0 (0)**	111	80	**191**	0 (0)	0 (0)	**0 (0)**
H	74	53	45	**172**	8 (10.8)	6 (11.3)	1 (2.2)	**15 (8.7)**	2 (2.7)	2 (3.8)	0 (0)	**4 (2.3)**	48	16	**64**	0 (0)	0 (0)	**0 (0)**
I	81	104	34	**219**	0 (0)	1 (0.96)	1 (2.9)	**2 (0.9)**	10 (12.3)	18 (17.3)	3 (8.8)	**31 (14.2)**	n. a.	n. a.	**n. a.**	n. a.	n. a.	**n. a.**
Total Leipzig	212	219	108	**539**	9 (4.3)	11 (5.0)	3 (2.8)	**22 (4.1) 1 x*Bcap*, 3 x*B*. sp. EU1, 18 x*Bm***	19 (9.0)	25 (11.4)	3 (2.8-3.7)	**47 (8.7)**	497	307	**804**	0 (0)	0 (0)	**0 (0)**
Bavaria	42	58	28	**128**	4 (9.5)	3 (5.2)	0 (0)	**7 (5.5) (2 x*Bd*, 2 x*B.*sp. EU1, 3 x*Bm*)**	1 (2.4)	10 (17.2)	1 (3.6)	**12 (9.4)**	95	40	**135**	0 (0)	0 (0)	**0 (0)**
Saarland^a^	43	36	36	**115**	2 (4.7)	4 (11.1)	1-2 (2.8-5.1)	**7 (6.1)**^**b**^**1 x*B.*sp. EU1, 4 x*Bm*, 2 x*Ba***	9 (21.0)	9 (25.0)	2-7 (5.6-19.4)	**20 (17.4)**^**b**^						

*Bcap*, *Babesia capreoli*; *Bd*, *Babesia divergens*; *B.* sp. EU1, *Babesia* sp. EU1; *Bm*, *Babesia microti; Ba, Babesia* sp.

^a^ results of the *D. reticulatus* from the Saarland have been published elsewhere [14].

^b^ minimum infection rate: nymphs were investigated in pools of up to five - assuming that only one tick in the pool was infected.

### Small mammals

Overall, 72 animals of 4 species were trapped: bank vole (*Myodes glareolus*; n = 36), yellow-necked mouse (*Apodemus flavicollis*; n = 32), and striped field mouse (*A. agrarius*; n = 3). One common shrew (*Sorex araneus*) was found dead in a trap. 21 animals were trapped at site E (16 in 2010; 5 in 2011; 14 bank voles, 6 yellow-necked mice, 1 common shrew), 21 animals at site I (17 in 2010; 4 in 2011; 14 bank voles, 7 yellow-necked mice), and 30 animals at site H (14 in 2010, 16 in 2011; 8 bank voles, 19 yellow-necked mice, 3 striped field mice). The average trapping success during the nine trapping days was 12% (2.5%-19%). A further 8 animals of three additional species captured by a cat at a different location within the renatured brown coal surface mining sites were investigated (* in Figure 1): water voles (*Arvicola terrestris*, n = 3); shrews (*Crocidura russula*, n = 4) and a common mole (*Talpa europaea*, n = 1). DNA from blood (n = 31), transudates (n = 80), spleens (n = 79), kidneys (n = 80) and ears (n = 80) were used for the molecular analysis in this study.

### Tick infestation on small mammals

Overall, 365 ticks were collected from 50 of the 80 small mammals. The infested mammals were 15 out of 31 females and 35 out of 49 males. Species identification revealed 232 *I. ricinus* (44 nymphs, 188 larvae) and 117 *D. reticulatus* (112 nymphs, 5 larvae) (Table 5). One *I. trianguliceps* larva was identified on a bank vole at site I. A further 12 ticks were identified as *Ixodes* spp., 1 as *Dermacentor* spp. and for 2, identification was not possible due to damage. *Ixodes* spp. were obtained on 6 small mammal species (except *S. araneus*) at all 3 trapping sites during all trapping months (Table 6). Comparable numbers of them had tick infestation (slightly more in yellow-necked mice), and of those, comparable numbers were infested with *I. ricinus* larvae and nymphs with comparable median numbers of ticks (Table 6). In contrast, *D. reticulatus* were obtained on bank voles only (apart from 1 nymph on one yellow-necked mouse), exclusively during the month of August and only at sites E and I, even though at the latter no questing *D. reticulatus* had been detected (Table 3). The median of *D. reticulatus* nymphs was higher than that of *I. ricinus* larvae and nymphs on bank voles. 95.4% (n = 21) out of the 22 tick-infested yellow-necked mice were infested with *I. ricinus* larvae, 36.4% with *I. ricinus* nymphs and 4.5% (1 individual) with a *D. reticulatus* nymph. 80.1% out of the 21 tick-infested bank voles were infested with *I. ricinus* larvae, 28.6% with *I. ricinus* nymphs, 4.8% with *D. reticulatus* larvae and 62% with *D. reticulatus* nymphs.

**Table 5:** **Small mammals trapped at recreational areas of Leipzig during 2010 and 2011: PCR results of the investigations for *Babesia * spp. and *Anaplasma phagocytophilum * and their tick infestation**^**a**^

		**No. Positive for**		**No. with (%)**		
**Small mammalian species**	**No.**	***Ap***	***Ba***	**ticks**	***Ir***	***Dr***
**Yellow-necked mouse**	32			22 (68.8)	22 (68.8)	1 (3.1)
**Striped field mouse**	3		1*Bm* (Ki, Tr; site H)	2 (66.7)	2 (66.7)	
**Bank vole**	36	2 (2 Tr, 1 Ki; site E)	1 *Hep* (Tr; site E)	21 (58.3)	17 (47.2)	13 (36.1)
**European water vole**	3			2 (66.7)	2 (66.7)	
**Common mole**	1		1 *B. *sp. EU1 (Tr; site^*^)	1 (100.0)	1 (100.0)	
**Greater white-toothed shrew**	4			2 (50.0)	2 (50.0)	
**Common shrew**	1		1 *Sar* (Tr; site E)			
**Total**	**80**	**2**	**2**^**b**^	**50**	**46**	**14**

*Ba*, *Babesia* spp.; *Ap*, *Anaplasma phagocytophilum; Ir, Ixodes ricinus; Dr, Dermacentor reticulatus; Bm, Babesia microti; B.*sp. EU1, *Babesia* sp. EU1; *Hep, Hepatozoon; Sar, Sarcocystis;* Ki, kidney; Tr, transsudate.

^a^only *I. ricinus* and *D. reticulatus* are shown; ^b^2 positive for *Babesia*, but altogether 4 positive in the PCR.

**Table 6:** Small mammals trapped at recreational areas of Leipzig during 2010 and 2011: detailed results of their tick infestation and results of the PCR screening of the ticks for *Babesia * spp. and Anaplasma phagocytophilum^*^

		**No. with infestation**										
		**[median no. of ticks (range)]**										
		**larvae**					**nymphs**					
**Small mammalian species**	**No.**	***Ir***	***It***	***Ix***	***Dr***	**unid*.***	***Ir***	***Ap*-positive**	***Ba*-positive**	***Dr***	***Derm.***	**unid*.***
**Yellow-necked mouse**	32	21 [2 (1–16)]		4 [2 (1–2)]		1 [1 (1)]	8 [1 (1–17)]	1 (I)	1 *B*. sp*.* EU1 (eng; site H)	1 [1 (1)]		1 [1 (1)]
**Striped field mouse**	3	2 [6.5 (1–12)]		1 [1 (1)]			1 [5 (5)]					
**Bank vole**	36	17 [3 (1–13)]	1 [1 (1)]	2 [1.5 (1–2)]	1 [5 ( 5)]		6 [1 (1–2)]	1 (E)	1 *B*. sp*.* EU1 (uneng; site I)	13 [7 (1–22)]	1 [1 (1)]	
**European water vole**	3						2 [1.5 (1–2)]					
**Common mole**	1	1 [4 (4)]										
**Greater white-toothed shrew**	4	2 [5.5 (2–9)]					1 [1 (1)]					
**Common shrew**	1											
**Total**	**80**	**43**	**1**	**7**	**1**		**18**	**2**	**2**	**14**	**1**	**1**

*Ba, Babesia* spp*.; B*.sp*.* EU1, *Babesia* sp. EU1; *Ap*, *Anaplasma phagocytophilum; Ir, Ixodes ricinus; It, Ixodes trianguliceps; Ix, Ixodes spp.;* unid., unidentified species; *Dr, Dermacentor reticulatus; Derm., Dermacentor spp.;* eng, engorged; uneng, unengorged.

*All *Ixodes*-ticks (larvae and nymphs) were screened for *Babesia* sp. and *Anaplasma phagocytophilum*, all *Dermacentor*-ticks (larvae and nymphs) for *Babesia* spp., however, in this table only the positive results are shown.

Co-infestation and thus co-feeding of *I. ricinus* nymphs and larvae was observed on 7 yellow-necked mice, 8 bank voles, 1 striped field mouse and 1 shrew (*C. russula*). One of those yellow-necked mice and 5 bank voles were additionally infested with *D. reticulatus* nymphs and one bank vole with *D. reticulatus* larvae. 3 bank voles carried *I. ricinus* larvae and *D. reticulatus* nymphs and 1 bank vole *I. ricinus* larvae and an *I. trianguliceps* larva. The rest were single infestations of *I. ricinus* larvae (14 yellow-necked mice, 6 bank voles, 1 striped field mouse, 1 great white-toothed shrew, 1 common mole), *I. ricinus* nymphs (1 yellow-necked mouse, 2 European water voles) and *D. reticulatus* nymphs (4 bank voles). DNA was extracted individually from all host-attached nymphs.

### PCR analysis for *Babesia* spp

#### Questing ticks

Overall, 4.1% of *I. ricinus* (22/539; 95% CI: 2.6 to 6.1) were positive for DNA of *Babesia* spp. in Leipzig (Table 4). Average infection rates were 3.4% (5/148; 95% CI: 1.1 to 7.7) at sites E-G (Lake Cospuden), 8.7% (15/172; 95% CI: 5.0 to 14.0) at site H and 0.9% (2/219 95% CI: 0.1 to 3.5) at site I. In Bavaria, 5.5% of *I. ricinus* (7/128; 95% CI: 2.2 to 10.9) were positive, and in the Saarland, 6.1% (7/115; 95% CI 2.5 to 12.1). No statistically significant differences were observed between sites or developmental stages. Sequencing revealed *B. microti* (n = 3) and *Babesia* sp. EU1 (n = 2) at site E, *B. microti* (n = 14) and *B. capreoli* (n = 1) at site H and *B. microti* (n = 1) and *Babesia* spp. EU1 (n = 1) at site I. In questing ticks in Bavaria, the detected species were *B. microti* (n = 3), *B. divergens* (n = 2) and *Babesia* sp. EU1 (n = 2) and in the Saarland *B. microti* (n = 4), *Babesia* spp. (n = 2) and *Babesia* sp. EU1 (n = 1). All 939 questing adult *D*. *reticulatus*, for which DNA was successfully extracted, were negative for *Babesia*-DNA.

### *Small mammals and host-attached ticks*

The *Babesia* spp.-PCR was positive for the kidney and transudate of one striped field mouse from site H (*B. microti*), for the ear tissue of the European mole (*Babesia* sp. EU1) and for the transudates of a bank vole and a shrew from site E (Table 5). The latter two PCR-products revealed greatest similarity (99%) to *Hepatozoon* spp. BV1 [GenBank AY600626] for the bank vole and to *Sarcocystis* spp. isolate 5 [GenBank AF513487] for the shrew. The blood and spleens were all negative.

In two host-attached *I. ricinus* nymphs DNA of *Babesia* sp. EU1 was detected, the host-attached larvae were all negative (Table 6). The juvenile stages of *D. reticulatus* from small mammals were negative for DNA of *Babesia* spp.

### *Sequence analysis*

All 495 bp sequences from *B. microti* were 100% similar amongst each other and 100% similar to *B. microti* from *M. glareolus* (Germany), *I. ricinus* (Belgium, Switzerland and Germany) and also from the recently published first European case of human babesiosis due to *B. microti* in Germany (isolate Jena/Germany) [GenBank: AB085191, AF231349, AY144692, GQ856653] [5]. For one *B. microti*, only 465 bp could be evaluated. The 409 bp sequences of *Babesia* sp. EU1 were 100% identical to each other and to *Babesia* sp. EU1 from *I. ricinus* (Belgium), from *I. persulcatus* (Russia) and from roe deer (France and Austria) [GenBank: JF776414, GQ856657, HQ830266, GU734773, JN543171]. The sequence from the nymph collected from *A. flavicollis* differed in 1 nt position. The *B. capreoli* sequence (468 bp) from site H was 100% identical in the amplified part to the recently described *B. capreoli* including two of the 3 *B. capreoli* typical nucleotide (nt) positions at 631 (G) and 663 (T) of the *18S rRNA* gene [GenBank: FJ944828] [26]. For *B. divergens* sequences from Bavaria 463 bp and 325 bp were available and they were 100% identical to *B. divergens* with nucleotides AA at positions 631 and 663 [FJ944826] as described [26].

### PCR analysis for *Anaplasma phagocytophilum*

#### Questing ticks

A total of 8.7% (47/539; 95% CI: 6.5 to 11.4) of *I. ricinus* were positive for *A. phagocytophilum* in Leipzig (Table 4). Average prevalences were 8.1% (12/148; 95% CI: 4.3 to 13.7) at site E-G (Lake Cospuden), 2.3% (4/172; 95% CI: 0.6 to 5.8) at site H and 14.2% (31/219; 95% CI: 9.8 to 19.4) at site I. Nymphs were significantly less often infected than adult ticks (p < 0.05) (Table 4). Significantly less *I. ricinus* were infected with *A. phagocytophilum* at site H compared to the site E in Leipzig (*p* = 0.01). A total of 9.4% (12/128; 95% CI: 4.9 to 15.8) and 17.4% (20/115; 95% CI for the minimal infection rate: 11.0-25.6) *I. ricinus* were also positive for *A. phagocytophilum*-DNA in Bavaria and the Saarland, respectively (Table 4). Looking at the regions under investigation, there was no difference in the overall infection rate between Bavaria and Leipzig, but the ticks in the Saarland were significantly more often infected (*p* = 0.01).

### *Small mammals and host-attached ticks*

All blood and tissue samples of the small mammals were negative for *A. phagocytophilum*-DNA, but it was detected in the transudates of 2 bank voles from site E (Table 5). From one of them the kidney sample was also positive. One *I. ricinus* nymph collected from a bank vole from site E and from a yellow-necked mouse from site I were positive for *A. phagocytophilum*, all other host-attached ticks were negative (Table 6).

### *Sequence analysis*

Sequencing of 13 partial *16S rRNA* gene sequences from questing ticks revealed 4 gene variants. Variant “A” was detected at site I (n = 7) and in the Saarland (n = 1), variant “S” in Bavaria (n = 1), variant “X” at site E (n = 1) and I (n = 1) and variant “Y” in the Saarland (n = 2). This nomenclature is not official but has been used previously for comparison of *16S rRNA* variants [21,23,24]. Variant “A” has previously been detected in ticks, hedgehogs, dogs, horses, a cat and a human patient [GenBank: JF893935, FJ829748, HM138366] [24,27-31]. Variant “S” has previously been detected for example in horses, dogs, roe deer, red deer and ibex [GenBank: JF893934, FJ829790, FJ812391-FJ812397] [23,24,28]. The potentially apathogenic variants “X” and “Y” [GenBank: FJ538288, FJ538289, FJ812405, FJ812406] seem to be typical for roe deer and goats [23,32].

### Co-infections

Co-infections of *Babesia* spp. and *A*. *phagocytophilum* were not detected in 539 questing *I. ricinus* in Leipzig and in 128 questing *I. ricinus* in Bavaria, whereas in the Saarland 1/115 (0.9%; one adult male) was infected with *B. microti* and *A. phagocytophilum*.

With regard to co-infections and differences in prevalence patterns we also statistically analysed data from a previous study in the same questing ticks which were investigated for *Rickettsia* spp. (*R. helvetica* in *I. ricinus* and *R. raoultii* in *D. reticulatus*) [13]. In Leipzig, 7/539 *I. ricinus* (1.3%; 3 adult male and one adult female from site I; one adult male and two females from site E) were coinfected with *R. helvetica* and *A. phagocytophilum*, and 1/539 (0.2%; one adult female) from site H was coinfected with *B. microti* and *R. helvetica*. In Bavaria, 1/128 *I. ricinus* (0.8%; one adult male) was coinfected with *R. helvetica* and *A. phagocytophilum*. In the Saarland, altogether 6 *I. ricinus* samples (5.2%; 4 males, 1 female, 1 nymphal pool containing two nymphs) were coinfected with *R. helvetica* and *A. phagocytophilum*, one adult female with *R. helvetica* and *B. microti* and one nymph pool (containing two nymphs) with *Babesia* spp. and *R. helvetica*. Triple infections were not detected [13]. The comparison of the single collection sites amongst each other revealed for *I. ricinus* a significant difference in the infection rates with *R. helvetica* between site E and I in Leipzig (p = 0.02). For *D. reticulatus*, the collection sites in the Saarland and Bavaria, as well as the site G revealed a lower prevalence compared to site E which was used as a reference site for the analysis. The comparison of the three regions showed significantly higher prevalences in *I. ricinus* in comparison to Leipzig for Bavaria (*p* = 0.001) and the Saarland (*p* < 0.001), whereas for *D. reticulatus*, the infection was significantly higher in Leipzig compared to Bavaria and the Saarland (for both *p* < 0.001).

## Discussion

To investigate the host-pathogen-vector interface in urban recreational areas in the city of Leipzig, Germany, questing *I. ricinus* and *D. reticulatus* ticks, small mammals and the ticks infesting them were screened by molecular methods for the presence of zoonotic *Babesia* spp. and *A. phagocytophilum*. Additionally, questing ticks from Bavaria and the Saarland were collected for comparative purposes. The questing tick collection revealed a coexistence of *I. ricinus* and *D. reticulatus* in recreationally used renatured areas in Leipzig. Reports on the existence of *D. reticulatus* in Eastern Germany date back to the first half of the 20^th^ century [33,34], but recently reports about their abundance in new or renewed habitats are accumulating [13,35-39]. Climatic change or changes in landscape structure or land use are being discussed for its geographical expansion and our findings in a newly created renatured area adds evidence to this [13,40].

### PCR results for *Babesia* spp

Average detection rates in questing *I. ricinus* ticks of DNA of *Babesia* spp. were 4.1% in Leipzig, 5.5% in Bavaria and 6.1% the Saarland) and the species *B. capreoli*, *B. divergens*, *B. microti*, *Babesia* sp. EU1 and *Babesia* spp. were identified by sequencing. We detected DNA of *B. microti* also in the kidney of only one *A. agrarius* but not in its other tissues. Previous PCR-detections of *B. microti* in small mammals in Europe ranged from 1.6% to 42% in microtine voles and from 1% to 11.8% in *A. flavicollis*[41-45] and generally voles seem to be infected more often with *B. microti* than mice [46]. Thus, voles and shrews, but not mice are considered as reservoirs [43]. These small mammalian species all live in urban environments, but the abundance of *Apodemus* spp. is considered to be higher than that of *M. glareolus*[46,47]. As expected, at site H in the present study (an urban park) more *Apodemus* spp. were trapped than voles (this was the opposite for sites E and I), nonetheless in questing *I. ricinus* ticks a high prevalence of *B. microti* was found at site H. This is a rather surprising result and shows that further systematic investigations in this geographic area are needed, but also that the choice of tissue for investigation has a strong influence on the outcome of a study.

The presence of *Babesia* sp. EU1 was shown for the first time in questing ticks in the Middle of Germany in the present study. The mammalian reservoir for *Babesia* sp. EU1 is unknown, but roe deer has also been suggested [8]. This may explain the predominance of this *Babesia* species around Lake Cospuden where roe deer has found a new habitat. We detected *Babesia* sp. EU1-DNA in two nymphs collected from rodents and in the ear tissue of a common mole, which is the first detection of this *Babesia* species in this insectivore. Whether this is a rare coincidence or whether *T. europaea*, or other rodents and insectivores, play a role in the life cycle of *Babesia* sp. EU1 remains to be investigated. Apicomplexan parasites as well as trypanosomes have been shown in the red blood cells of the common mole before [48,49]. Isolation of the tick-borne encephalitis virus was achieved from a common mole, showing that this insectivore species may indeed play a role in the life cycle of some tick-borne pathogens [50]. It is interesting in this context that we are not aware of any description of *Babesia* spp. other than *B. microti* in small mammals thus far. We cannot tell whether this is because other investigators specifically look for *B. microti* or do not differentiate the *Babesia* spp., but the *Babesia* sp. EU1 detection in the European mole is a new and interesting finding in this regard. The detection of three zoonotic *Babesia* species in actively questing *I. ricinus* in recreational areas in Germany has considerable public health implications even though, in Europe, human babesiosis occurs almost exclusively in splenectomised or otherwise immunocompromised individuals [4,11].

In the present study, no evidence was found in the investigated *D. reticulatus* populations in Leipzig and Bavaria for infection with *B. canis* even though reports on autochthonous cases of canine babesiosis occur occasionally in Germany [51]. Discussion is ongoing that the import of persistently infected dogs may provide a source and could contribute to the creation of new endemic cycles in previously *Babesia*-free *D. reticulatus* populations [52].

It is noteworthy that both a *Hepatozoon* spp. and a *Sarcocystis* spp. were identified in a bank vole and a shrew due to two reasons, respectively. Firstly, this shows that in an apparently genus-specific PCR other genera of closely related protozoa may also be amplified and highlights the importance of sequencing in such screenings. In our particular case, this would have resulted in doubling the prevalence of *Babesia* spp. in the small mammals investigated. Secondly and even more importantly, it also shows that rodents and insectivores are hosts to a very large parasitical and bacterial fauna.

### PCR results for *Anaplasma phagocytophilum*

The average prevalence of *A. phagocytophilum* in *Ixodes ricinus* in the investigated areas in Leipzig (8.7%) was considerably higher than most previously detected average infection rates in Germany (see Table 2) and was most similar to average prevalences recorded from city parks in Bavaria [29,53]. The presence of a different reservoir host had been hypothesised as a reason for this in areas with a city park character. However, in Leipzig, a city park (Site H) had the lowest prevalence (2.3%). The highest prevalence (14.2%) was observed close to the game park (site I). The presence of cervids might lead to a higher prevalence of *A. phagocytophilum* in ticks, as for example roe deer is frequently infested with ticks and is discussed as a potential reservoir host [12,54]. Similarly high prevalences of up to 17.1% have been detected in Norway in sites with a high density of wild cervids [55]. Surprisingly, and also on the contrary to the “city park” hypothesis, an infection rate of 9.4% was recognized in the alluvial forest north of Munich along the river Isar. The prevalence in an earlier study in the same region was remarkably lower (females 4.1%, males 0.75%) [29]. The difference in prevalence rates may also be based on a year-to-year fluctuation, as has been shown in other studies [56,57]. For *Borreli*a spp., a correlation was observed between peak abundance of rodent populations and an increase of infection in ticks in following years [58].

Significantly less nymphs than adults were infected with *A. phagocytophilum* in the present study. This result is well explained by the lack of transovarial transmission of *A. phagocytophilum* and has been shown in previous studies as well [29,53]. Apart from roe deer, small mammals have been suggested as reservoir hosts for *A. phagocytophilum*. In the present study, transudates of two bank voles were positive for DNA of *A. phagocytophilum* as well as one kidney sample. In the U.K., where *A. phagocytophilum* was found in field voles, bank voles and wood mice, the infection rate was generally higher in voles. Higher infection was further observed in sites with occurrence of *I. trianguliceps*. Rodents were not or less infected in sites where only *I. ricinus* occurred [59,60]. A separate endemic niche cycle involving voles and *I. trianguliceps* was suggested for *A. phagocytophilum*[59-61]. When individual voles were investigated several times during one study, *A. phagocytophilum* infection seemed short-lived whereas rodents were persistently infected with *B. microti*[45,59]. Only voles were infected with *A. phagocytophilum* in this study which coincides with this hypothesis. This could be the reason for the low prevalence with *A. phagocytophilum* in this study.

Larvae are free of *A. phagocytophilum* as no transovarial transmission occurs and adult ticks do not generally feed on rodents. Consequently, larvae need to acquire the infection from rodents and following this, nymphs have to give it back to the rodents to keep up the endemic cycle. Keeping in mind that small rodents are themselves short-lived animals and that the infection with *A. phagocytophilum* is transient, the question has to be raised if they would provide a suitable reservoir for keeping an endemic cycle. We observed a high percentage of co-feeding ticks on the trapped small mammals and similar to *Borrelia* spp. or the TBE-virus, this could be part of the endemic cycle [62,63].

### *Anaplasma phagocytophilum 16S rRNA* gene variants

Four genetic variants of the partial *16S rRNA* of *A. phagocytophilum* were detected in questing ticks in this study, all of which have been previously described. Interestingly, the human pathogenic prototype variant “B” [equalling GenBank U02521 in the amplified part] was not detected. About one-fourth of the *A. phagocytophilum* positive samples were sequenced and the variant “A” was the most prevalent variant (8 of 13). This variant is very common in ticks in Germany and has also been detected as the most common variant in hedgehogs [29,30,53]. Variant “A” is also known to cause granulocytic anaplasmosis in humans, horses and dogs, but apparently to a lesser extent [24,27,28,31]. Variant “S” has been previously detected in clinical cases of dogs and horses [24,28]. Variants “X” and “Y” seem to be typical for caprines and cervids, and have also been previously detected in ticks [23,27,32,53]. Due to the low occurrence of seemingly pathogenic variants, the high prevalences and the resulting actual risk for human beings to develop clinical disease has to be carefully evaluated. In the USA, human granulocytic anaplasmosis (HGA) is the second most important tick-borne disease after Lyme Borreliosis, whereas in Europe only about 70 cases have been reported [64]. Even though human cases have to date not been reported in Germany, seroprevalence studies have shown that the human population is exposed to the agent [65,66] and granulocytic anaplasmosis cases have been reported from dogs and horses [67-69]. Around Germany, HGA cases have been reported, for example from Slovenia, Austria and Poland [70-72].

### Co-infections

The rates of co-infections detected in this study were less than 1% of investigated ticks and similar to previously detected ones [18]. The lowest co-infection rate seems to occur between *Babesia* spp. and *Rickettsia* spp., and the highest between *Rickettsia* spp. and *A. phagocytophilum*[18]. The co-infection rate of *Rickettsia* spp. and *A. phagocytophilum* was especially high in the Saarland (5.2%), and can be lead back to the fact that 17.4% *I. ricinus* ticks were infected with *A. phagocytophilum*. Co-infections between the pathogens investigated in this study have also been detected in other European countries [73-80].

### Tick infestation on small mammals

Developmental stages of *D. reticulatus* were observed almost exclusively on bank voles. Similar results were obtained previously [62,81]. Furthermore, they were only observed during trapping in the month of August. This reflects the seasonal dynamics of this tick species, with developmental stages being active during the months of July and August [16], whereas adults have their activity peak in spring and autumn [15]. The developmental stages have an endophilic life cycle adapted to their small mammalian hosts and are therefore not obtained by the flagging method.

Co-feeding of both developmental stages of *I. ricinus* and additionally co-infestation with both hard tick species was frequently observed, especially in bank voles. This finding highlights the importance of rodent species in the developmental cycles of ticks and consequently for tick-borne pathogens. Larvae and nymphs of *I. ricinus* originate most probably from different tick generations, based on the life cycle of this tick species, whereas *Dermacentor* spp. can complete its entire life cycle within one year [16] which may result in different dynamics of the involved pathogens. *I. ricinus* is the main vector in Europe for *A. phagocytophilum*, *R. helvetica* and the *Babesia* species detected in this study. Even though *I. trianguliceps* may also act as a vector for *A. phagocytophilum*, its occurrence is relatively rare in Central and Eastern Europe, and therefore its importance is less apparent [59]. Furthermore, *I. trianguliceps* rarely feeds on humans whereas this is regularly the case for *I. ricinus*. In contrast, the distribution of *D. reticulatus* is highly focally. Its developmental stages do not attack humans, and the adult stages only rarely do. Therefore the public health risk of *I. ricinus*-borne pathogens has to be considered way higher by comparison, especially as co-infections with several pathogens can be detected. However, *D. reticulatus* is a tick frequently observed on dogs, thus posing a health threat for the canine population.

## Conclusion

In conclusion, three zoonotic *Babesia* species and *A. phagocytophilum* as well as co-infections between the two and with *Rickettsia* spp. were detected in three areas of Germany – Leipzig, north of Munich and the Saarland. As the areas under investigation are recreational, the detection of *A. phagocytophilum* has both implications for human and animal health and the detection of the zoonotic *Babesia* spp. is of importance for humans. Although we provide new data regarding the distribution patterns of the described pathogens, we still lack a thorough understanding of the epidemiology and interplay of the ticks, their feeding sources and pathogens. In particular, knowledge about the role of co-feeding in the transmission cycles, as well as the specific hosts and potential reservoir animals in these areas, is the essential basis for the development of effective preventive control measures.

## Competing interests

The authors declare that they have no conflict of interest of any kind.

## Authors’ contributions

CS and MP conceived the idea and design of the study. CS, DW, MP and DH carried out field and laboratory work. CS analysed the data and wrote the manuscript. MM performed the statistical analysis. MP and KP critically revised the manuscript. All authors read and approved the final version of the manuscript.
